# Functional disability and multidimensional hardship among cancer survivors in the USA: a nationally representative study

**DOI:** 10.1007/s11764-026-01981-y

**Published:** 2026-02-12

**Authors:** Areesh Mevawalla, Odysseas P. Chatzipanagiotou, Azza Sarfraz, Timothy M. Pawlik

**Affiliations:** Department of Surgery, The Ohio State University Wexner Medical Center and James Comprehensive Cancer Center, 395 W. 12thAve.Suite 670, Columbus, OH USA

**Keywords:** BRFSS, Cancer survivorship, Disability, Health disparities, Functional limitation, Medicaid expansion

## Abstract

**Purpose:**

Functional disability is common among cancer survivors, yet its contribution to multidimensional survivorship burden remains incompletely characterized. We evaluated disability-specific and sex-stratified associations with survivorship burden in a nationally representative US cohort.

**Methods:**

We analyzed 2017–2022 Behavioral Risk Factor Surveillance System data among adult cancer survivors. Functional disability included mobility, cognitive, or self-care limitation. Outcomes included socioeconomic hardship (ordinal composite of inability to work and cost-related unmet care), psychological burden (frequent mental distress), and psychosocial burden (leisure-time physical inactivity). Survey-weighted regression models assessed associations by disability burden and type, with sex-stratified analyses and disability-by-sex interaction testing. Difference-in-differences analyses evaluated disability-associated socioeconomic hardship by Medicaid expansion status among low-income survivors.

**Results:**

Among 3.57 million weighted cancer survivors, mobility (28.0%), cognitive (12.9%), and self-care (6.4%) disabilities were prevalent. Any disability was associated with higher socioeconomic hardship (aPOR 2.48, 95% CI 2.22–2.77), psychological burden (aOR 2.54, 95% CI 2.33–2.77), and psychosocial burden (aOR 2.68, 95% CI 2.48–2.89). Disability-specific patterns differed: cognitive disability was most strongly associated with psychological burden (aOR 3.42, 95% CI 3.02–3.87), self-care disability with socioeconomic hardship (aPOR 2.63, 95% CI 2.12–3.27), and mobility disability with psychosocial burden (aOR 2.98, 95% CI 2.70–3.29). Medicaid expansion modestly attenuated disability-associated socioeconomic hardship among low-income survivors. Disability-associated socioeconomic and psychological burden was greater among women, whereas psychosocial burden was more pronounced among men.

**Conclusion:**

Functional disability was strongly associated with multidimensional survivorship burden, with distinct disability- and sex-specific patterns.

**Implications for Cancer Survivors:**

Disability-informed survivorship care may reduce hardship and improve post-cancer quality of life.

**Supplementary Information:**

The online version contains supplementary material available at https://doi.org/10.1007/s11764-026-01981-y.

## Introduction

Cancer survivorship in the USA has risen substantially in recent decades. As of 2025, approximately 18.6 million Americans are living with a history of cancer, a number expected to surpass 22 million by 2035 [1]. Advances in early detection and treatment have prolonged survival, allowing more individuals to live longer following a cancer diagnosis [2]. However, many cancer survivors face long-term physical and cognitive sequelae, such as cardiopulmonary complications and “chemo brain,” that can impair daily functioning [2]. As such, functional disability has emerged as a key concern in the growing survivor population.

Recent studies indicate that nearly two-thirds of US cancer survivors experience difficulties with routine activities—a rate almost twice that of the general population [3]. Approximately 30% of survivors report serious difficulty walking or climbing stairs, and 7.4% struggle with self-care tasks like dressing or bathing [4]. In addition, nearly half of cancer survivors experience persistent cognitive challenges affecting memory, concentration, and other mental functions [5]. These functional deficits are strongly linked to reduced quality of life and greater hardship among survivors [6]. Despite these concerns, there remains a notable gap in the literature on the full extent and impact of mobility, cognitive, and self-care disabilities among US cancer survivors, particularly regarding their associations with employment disruption, barriers to medical care, and mental health distress [7–9].

The aforementioned patterns highlight the need not only to prolong survival after cancer, but also to improve survivors’ quality of life [3]. However, to date, few studies have comprehensively assessed the burden of specific types of disability or examined their downstream effects on cancer survivors’ well-being. Therefore, the objective of the current study was to evaluate the prevalence and impact of functional disability among US cancer survivors between 2017 and 2022. Disability-specific associations with socioeconomic hardship, defined as a composite of employment disruption and cost-related barriers to care, were evaluated; in addition, psychological burden and psychosocial burden, reflected by mental health distress and leisure-time physical inactivity, were characterized.

## Methods

### Data source, cohort selection, and outcomes

This cross-sectional analysis used pooled data from the 2017–2022 Behavioral Risk Factor Surveillance System (BRFSS), an annual, state-based telephone survey administered by the Centers for Disease Control and Prevention that samples non-institutionalized US adults aged ≥ 18 years [10]. BRFSS employs a complex, multistage probability design; all estimates were calculated with the CDC-supplied sampling weights, strata, and primary sampling units to yield nationally representative results. Among 2,591,503 respondents in the analytic sample, cancer survivors were identified using self-reported cancer diagnosis, including breast, lung, colorectal (colon and rectal), prostate, cervical, ovarian, endometrial, kidney, liver, stomach, esophageal, thyroid, and brain cancers, consistent with BRFSS cancer history classifications. We excluded respondents with incomplete state residence data (necessary to apply survey weights correctly) and individuals with missing responses on all four primary survivorship outcomes (unable to work, ≥ 14 days of poor mental health, cost-related unmet medical need, no leisure-time physical activity), as their hardship and burden status could not be assessed.

Functional disability was assessed based on three standardized BRFSS questions, each recoded as a binary indicator (1 = “yes”, 0 = “no”). These questions focused on cognitive disability (“Because of a physical, mental, or emotional condition, do you have serious difficulty concentrating, remembering, or making decisions?”), mobility disability (“Do you have serious difficulty walking or climbing stairs?”), and self-care disability (“Do you have difficulty dressing or bathing?”). A disability count variable was created by summing the three indicators that was categorized as 0, 1, 2, or 3 disabilities. In addition, for disability-type–specific analyses, mutually exclusive disability categories were defined, including no disability (reference), mobility only, cognitive only, and self-care only; individuals reporting more than one disability were excluded from these type-specific comparisons.

The primary outcomes of interest encompassed three domains of survivorship burden: (1) socioeconomic hardship, defined as two indicators—self-reported inability to work because of health and cost-related delay in medical care—each assessed individually and jointly as an ordinal composite (0, 1, or 2 hardships) [11–15]; (2) psychological burden, defined as experiencing ≥ 14 days of poor mental health in the past 30 days [16]; and (3) psychosocial burden, defined as no reported leisure-time physical activity in the past month [17, 18]. The socioeconomic hardship composite was constructed to capture cumulative material and employment-related vulnerability among cancer survivors, consistent with prior survivorship and health services research [19, 20]. Psychological and psychosocial outcomes were examined as distinct but complementary dimensions of survivorship burden reflecting mental health distress and reduced engagement in health-promoting behaviors [21, 22]. This study used publicly available, de-identified data and was deemed exempt by The Ohio State University Institutional Review Board.

### Covariates

Demographic covariates included age (< 65, ≥ 65 years), sex assigned at birth (male, female), and race/ethnicity (White, Black, Hispanic, Asian, Native American, Other). Educational attainment was categorized as less than high school, high school diploma or some college, and college degree or above. Residence was classified as urban or rural, and marital status as married or not married. Annual household income was grouped as < $25,000, $25,000–$74,999, and ≥ $75,000 [23]. Health-related covariates included age-stratified health insurance status (ages 18–64: private, Medicaid, or other; ages ≥ 65: Medicare, private, or other), presence of comorbidities (diabetes, heart disease, chronic obstructive pulmonary disease, stroke, and arthritis), and age at cancer diagnosis (< 40, 40–50, 51–64, ≥ 65 years). Access to care variables comprised having had a primary care visit in the past year and living in a Medicaid-expansion state.

### Statistical analysis

Descriptive statistics for categorical variables were presented as frequencies and percentages (%) and were analyzed using the Chi-square test or Fisher’s exact test, as appropriate. Continuous variables were reported as median values with interquartile ranges (IQR), with comparisons between groups conducted using the Kruskal–Wallis test. All weighting, clustering, and stratification variables supplied by BRFSS were applied in accordance with CDC guidance to generate nationally representative estimates. Associations between functional disability and survivorship outcomes were examined using survey-weighted multivariable regression models, with disability burden modeled as any disability (≥ 1 vs none) and by disability count (1, 2, or 3 vs 0). Socioeconomic hardship—defined as the ordinal sum (0, 1, or 2) of inability to work due to health and cost-related delay in medical care—was analyzed using ordinal logistic regression, while psychological burden (frequent mental distress) and psychosocial burden (leisure-time physical inactivity) were analyzed using multivariable logistic regression. Additional models examined mutually exclusive disability-type patterns (no disability, mobility only, cognitive only, self-care only). Sex-stratified analyses were conducted to evaluate differences in the association between disability burden and survivorship outcomes among female and male cancer survivors, with effect modification assessed using multiplicative disability-by-sex interaction terms. Adjusted associations were estimated using survey-weighted regression models, and interaction *p*-values were derived from Wald tests. To assess the policy-level impact of Medicaid expansion on disability-associated socioeconomic hardship, analyses were restricted to low-income, working-age cancer survivors aged 18–64 years. For the difference-in-differences analyses, socioeconomic hardship was operationalized as a binary indicator (any hardship vs none; ≥ 1 of the two components) to estimate absolute risk differences in percentage points. A difference-in-differences framework was used to compare changes in hardship prevalence between Medicaid expansion and non-expansion states by disability type. Disability gaps were calculated as the difference in hardship prevalence among survivors with a given disability type versus individuals without disability within each state group; difference-in-differences estimates reflected the net change in disability-associated hardship attributable to Medicaid expansion. Statistical significance was assessed using two-sided tests with *p* < 0.05. All models were adjusted for the full set of covariates described above, variables with > 15% missing data were excluded from regression analyses, all tests were two-sided, and *p*-values < 0.05 were considered statistically significant. All analyses were conducted using Stata version 18 (StataCorp, College Station, TX).

## Results

### Baseline characteristics

**Fig. 1:**
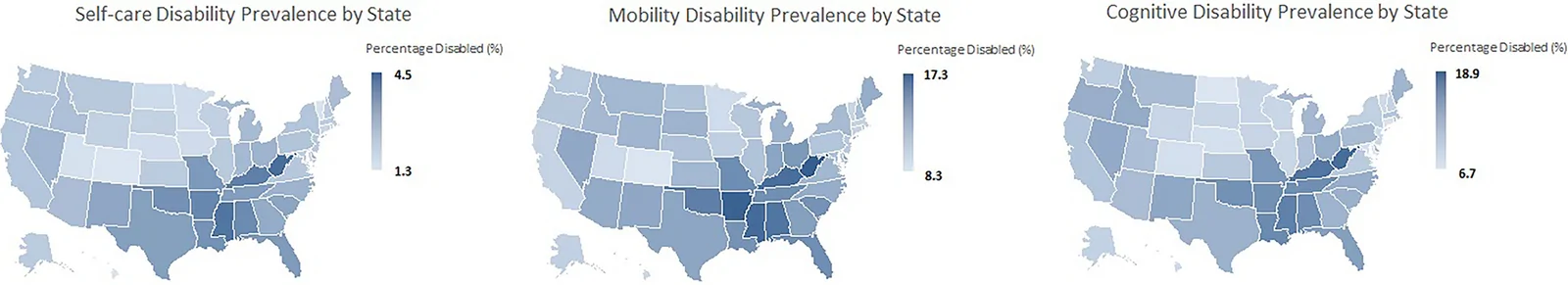
Geographic variation in functional disability prevalence among US cancer survivors, 2017–2022

Among 2,591,503 respondents included in the unweighted sample, 26,864 (1.0%) were self-reported cancer survivors. Cancer survivors were older than the general population (62 years [IQR 53–73] vs. 58 years [IQR 41–69). Cancer survivors were more frequently female (65.6% vs. 53.8), White (81.3% vs. 75.7%), and rural residents (26.4% vs. 23.0%; *p* < 0.001), compared with the general population (all *p* < 0.001). Of note, a higher prevalence of self-care, mobility, and cognitive disabilities was observed in the Southeast and Appalachian states, whereas rates were markedly lower across the Mountain West and Upper Midwest (Fig. 1). Overall, functional limitations were much more common among cancer survivors than the general population, including mobility disability (28.0% vs. 15.9%), cognitive disability (12.9% vs 10.5%), and self-care disability (6.4% vs 4.0%) (all *p* < 0.001, eTable 1).

Following application of survey weights, the estimated cancer survivor cohort included 3,570,518 adults (Table 1). Overall, 2,012,487 survivors (56.4%) reported no functional disability. Among individuals with a disability, 445,912 (12.5%) had mobility disability only, 300,418 (8.4%) cognitive disability only, and 171,034 (4.8%) self-care disability only, while 640,667 survivors (17.9%) reported two or more disability domains (eTable 2). Survivors with higher disability burden were older and more frequently female, with 395,044 individuals aged ≥ 65 years (86.5%) among those individuals with two disabilities and 157,937 (85.9%) among individuals with three disabilities (*p* < 0.001, Table 1). Lower household income (< $25,000) was increasingly common with greater disability burden, reported by 153,487 survivors (7.6%) with no disability, 132,119 (14.4%) with one disability, 105,763 (23.1%) with two disabilities, and 53,383 (29.0%) with three disabilities (*p* < 0.001). Primary care utilization remained high across all groups but declined modestly with increasing disability count, with recent primary care visits reported by 1,793,914 survivors (89.1%) without disability, 827,663 (90.2%) with one disability, 417,116 (91.3%) with two disabilities, and 164,479 (89.5%) with three disabilities (*p* < 0.001). Distributions of cancer type across disability burden categories are detailed in Table 1.

**Table 1:** Weighted characteristics of cancer survivors by disability domain

**Characteristics**	**All cancer survivors **(*n* = 3,570,518)	**0 disabilities **(*n* = 2,012,487 (56.4%)	**1 disability **(*n* = 917,364 (25.7%)	**2 disabilities **(*n* = 456,902 (12.8%)	**3 disabilities **(*n* = 183,765 (5.1%)	***p* value**
**Age, years**						< 0.001
< 65	1,159,893 (32.4)	890,126 (44.2)	191,433 (20.9)	57,016 (12.5)	21,318 (11.6)	
> 65	2,372,853 (66.4)	1,100,992 (54.7)	718,880 (78.4)	395,044 (86.5)	157,937 (85.9)	
**Sex**						< 0.001
Male	1,228,258 (34.4)	786,882 (39.1)	287,902 (31.4)	108,245 (23.7)	45,229 (24.6)	
Female	2,342,259 (65.6)	1,225,605 (60.9)	629,462 (68.6)	348,657 (76.3)	138,535 (75.4)	
**Ethnicity**						< 0.001
White	2,903,656 (81.3)	1,742,630 (86.6)	728,907 (79.5)	307,917 (67.4)	124,202 (67.6)	
Black	233,149 (6.5)	104,146 (5.2)	67,247 (7.3)	42,498 (9.3)	19,258 (10.5)	
Asian	66,766 (1.8)	42,534 (2.1)	15,221 (1.7)	6,201 (1.4)	2810 (1.5)	
Native American	71,953 (2.0)	28,917 (1.4)	21,847 (2.4)	13,853 (3.0)	7336 (4.0)	
Hispanic	190,988 (5.3)	78,812 (3.9)	55,226 (6.0)	38,183 (8.4)	18,767 (10.2)	
Other	106,400 (3.0)	15,448 (0.8)	29,616 (3.2)	48,250 (10.6)	13,086 (7.1)	
**Area**						< 0.001
Urban	2,116,562 (59.3)	1,279,944 (63.6)	527,481 (57.5)	227,998 (49.9)	81,139 (44.1)	
Rural	942,039 (26.4)	444,808 (22.1)	263,037 (28.7)	151,374 (33.1)	82,820 (45.1)	
**Level of education**						< 0.001
Less than high school	207,879 (5.8)	55,000 (2.7)	50,000 (5.5)	60,000 (13.1)	42,879 (23.3)	
High school/some college	1,945,125 (54.5)	976,707 (48.5)	545,000 (59.4)	310,125 (67.9)	113,293 (61.7)	
≥ College degree	1,412,593 (39.6)	980,000 (48.7)	320,000 (34.9)	85,000 (18.6)	27,593 (15.0)	
**Annual household income ($)**						< 0.001
< 25, 000	444,752 (12.4)	153,487 (7.6)	132,119 (14.4)	105,763 (23.1)	53,383 (29.0)	
25,000–74,999	717,668 (20.1)	428,931 (21.3)	209,774 (22.9)	66,908 (14.6)	12,055 (6.6)	
> 75,000	462,707 (12.9)	329,812 (16.4)	94,862 (10.3)	30,641 (6.7)	7392 (4.0)	
**Marital status**						< 0.001
Married	1,825,425 (51.1)	1,114,187 (55.4)	449,508 (49.0)	191,899 (41.9)	69,831 (38.0)	
**Comorbidities**						< 0.001
Diabetes	1,297,382 (36.3)	705,913 (35.1)	333,847 (36.4)	180,291 (39.5)	77,331 (42.1)	
Heart disease	367,878 (10.3)	160,429 (8.0)	96,118 (10.5)	69,905 (15.3)	41,426 (22.5)	
COPD	525,616 (14.7)	240,876 (12.0)	142,503 (15.5)	98,214 (21.5)	44,023 (24.0)	
Stroke	258,685 (7.2)	108,912 (5.4)	70,488 (7.7)	50,961 (11.2)	28,324 (15.4)	
Arthritis	1,849,365 (51.8)	875,214 (43.5)	515,306 (56.2)	304,587 (66.7)	154,258 (83.9)	
**Age at diagnosis, years**						< 0.001
≥ 65	563,255 (15.8)	261,623 (13.0)	146,778 (15.9)	91,380 (20.0)	44,104 (24.0)	
51–64	649,705 (18.2)	362,248 (18.1)	165,126 (17.8)	86,711 (18.9)	36,753 (20.0)	
40–50	1,213,625 (34.0)	704,371 (34.8)	311,904 (33.9)	140,383 (30.7)	56,967 (31.0)	
< 40	1,146,327 (32.1)	684,245 (34.0)	293,556 (32.0)	122,585 (26.8)	45,941 (25.0)	
**Treatment status**						
Completed	470,421 (13.2)	215,904 (10.7)	129,844 (14.2)	82,316 (18.0)	42,357 (23.0)	< 0.001
**Primary care visit**						
Had PCP visit (past year)	3,203,172 (89.7)	1,793,914 (89.1)	827,663 (90.2)	417,116 (91.3)	164,479 (89.5)	< 0.001
**Health insurance status**						
**Age 18–64**						
Private	826,512 (23.1)	676,520 (76.1)	116,874 (60.8)	25,658 (44.7)	7460 (35.0)	< 0.001
Medicaid	247,652 (6.9)	151,321 (17.2)	58,953 (30.8)	25,658 (45.0)	11,720 (55.0)	
Other	85,729 (2.4)	62,285 (6.9)	15,606 (8.2)	5700 (10.0)	2138 (10.0)	
**Age > 65 years**						< 0.001
Medicare	1,864,489 (52.2)	803,724 (73.0%)	576,104 (80.1%)	340,939 (16.3%)	143,722 (91.0%)	
Private	381,538 (10.7)	231,208 (21.0%)	102,929 (14.3%)	39,504 (10.0%)	7897 (5.0%)	
Other	126,826 (3.6)	66,060 (6.0%)	39,847 (5.5%)	14,601 (3.7%)	6318 (4.0%)	
**State Medicaid Expansion Status**						
Yes	2,908,577 (81.5)	1,659,842 (82.5)	749,318 (81.7)	360,477 (78.9)	138,940 (75.6)	< 0.001
**Cancer types**						
Brain	26,467 (0.7)	12,418 (0.6)	6934 (0.8)	4287 (0.9)	2828 (1.5)	< 0.001
Breast	1,265,628 (35.4)	769,483 (38.2)	324,116 (35.3)	118,492 (25.9)	53,537 (29.1)	< 0.001
Lung	183,407 (5.1)	74,219 (3.7)	49,356 (5.4)	37,948 (8.3)	21,884 (11.9)	< 0.001
Thyroid	186,732 (5.21)	122,146 (6.1)	46,998 (5.1)	12,984 (2.8	4604 (2.5)	< 0.001
Esophagus	25,403 (0.7)	10,786 (0.5)	7012 (0.8)	5143 (1.1)	2462 (1.3)	< 0.001
Stomach	27,398 (0.9)	11,694 (0.6)	7528 (0.8)	5506 (1.2)	2670 (1.5)	< 0.001
Liver	34,447 (1.0)	14,126 (0.7)	9713 (1.1)	6543 (1.4)	4065 (2.2)	< 0.001
Colon	310,156 (8.6)	161,372 (8.0)	82,941 (9.0)	45,089 (9.8)	20,754 (11.3)	< 0.001
Rectal	32,585 (1.1)	16,943 (0.8)	8881 (1.0)	4667 (1.0)	2094 (1.1)	< 0.001
Kidney	119,833 (3.3)	62,487 (3.1)	31,616 (3.4)	17,982 (3.9)	7,748 (4.2)	< 0.001
Cervical	269,591 (7.5)	112,734 (5.6)	71,975 (7.9)	57,409 (12.6)	27,473 (15.0)	< 0.001
Ovarian	109,858 (3.0)	49,726 (2.5)	29,498 (3.2)	19,164 (4.2)	11,470 (6.2)	< 0.001
Endometrial	179,018 (5.0)	79,164 (3.9)	47,981 (5.2)	35,907 (7.9)	15,966 (8.7)	< 0.001
Prostate	789,621 (22.1)	530,274 (26.3	175,492 (19.1)	61,814 (13.5)	22,041 (12.0)	< 0.001

^1^Mobility disability defined as self-reported serious difficulty walking or climbing stairs

^2^Cognitive disability defined as serious difficulty concentrating, remembering, or making decisions due to a physical, mental, or emotional condition

^3^Self-care disability defined as difficulty dressing or bathing

### Association Between Disability Burden and Multidimensional Survivorship Burden

In adjusted analyses, cancer survivors reporting any functional disability had substantially higher burden across all survivorship domains compared with individuals without disability (Table 2). Specifically, the presence of any disability was associated with a 2.48-fold higher odds of greater socioeconomic hardship severity (aPOR 2.48, 95% CI 2.22–2.77), a 2.54-fold higher odds of psychological burden (frequent mental distress; aOR 2.54, 95% CI 2.33–2.77), and a 2.68-fold higher odds of psychosocial burden (leisure-time physical inactivity; aOR 2.68, 95% CI 2.48–2.89) (all *p* < 0.001) **(**Table 2**)**.

**Table 2:** Adjusted associations between disability burden and multidimensional hardship domains

Panel A. Primary exposure: any disability (≥ 1) vs none				
Outcome	**Model**	**Any disability vs none**		
Socioeconomic hardship (0/1/2)	Ordinal logit (aPOR)	2.48 (2.22–2.77)		
Psychological burden (frequent mental distress)	Logit (aOR)	2.54 (2.33–2.77)		
Psychosocial burden (leisure-time physical inactivity)	Logit (aOR)	2.68 (2.48–2.89)		
Panel B. Secondary exposure: Disability count (1/2/3 vs 0)				
Outcome	**Model**	**1 vs 0**	**2 vs 0**	**3 vs 0**
Socioeconomic hardship (0/1/2)	Ordinal logit (aPOR)	1.82 (1.62–2.05)	2.86 (2.51–3.26)	4.55 (3.85–5.37)
Psychological burden (frequent mental distress)	Logit (aOR)	1.79 (1.63–1.97)	2.92 (2.61–3.26)	4.41 (3.85–5.05)
Psychosocial burden (leisure-time physical inactivity)	Logit (aOR)	1.92 (1.77–2.08)	3.06 (2.78–3.37)	4.64 (4.10–5.25)

Abbreviations: *aOR*, adjusted odds ratio; *aPOR*, adjusted proportional odds ratio; *CI*, confidence interval; *BRFSS*, Behavioral Risk Factor Surveillance System

1. Exposure definitions: “Any disability” indicates reporting ≥ 1 functional disability domain (mobility, cognitive, or self-care). Disability count reflects the number of disability domains reported (1, 2, or 3), with 0 disabilities as the reference group

2. Outcome definitions:

• Socioeconomic hardship was defined as the sum of two indicators—(i) inability to work due to health and (ii) cost-related delay in medical care—categorized as 0, 1, or 2 hardships and modeled using ordinal logistic regression (aPOR)

• Psychological burden was assessed as frequent mental distress (≥ 14 days of poor mental health in the past 30 days)

• Psychosocial burden was operationalized as leisure-time physical inactivity (no leisure-time exercise/physical activity)

3. Covariate adjustment: Models were adjusted for all covariates listed in Table 1 , including age, sex, race/ethnicity, educational attainment, household income, marital status, residence (urban/rural), health insurance status, comorbidities (diabetes, heart disease, COPD, stroke, arthritis), primary care visit in the past year, treatment status, age at cancer diagnosis, state Medicaid expansion status, and cancer type

4. Survey design: All models incorporated BRFSS survey weights and design variables to produce nationally representative estimates

5. Ordinal model assumption: The proportional odds assumption for ordinal logistic regression was assessed (and met)

When disability burden was examined by the number of disability domains reported, a clear dose–response relationship was observed across all burden domains. Compared with survivors without disability, individuals reporting one disability had higher odds of socioeconomic hardship (aPOR 1.82, 95% CI 1.62–2.05), psychological burden (aOR 1.79, 95% CI 1.63–1.97), and psychosocial burden (aOR 1.92, 95% CI 1.77–2.08). These associations strengthened progressively among survivors reporting two disabilities (socioeconomic hardship: aPOR 2.86, 95% CI 2.51–3.26; psychological burden: aOR 2.92, 95% CI 2.61–3.26; psychosocial burden: aOR 3.06, 95% CI 2.78–3.37) and were most pronounced among survivors reporting three disability domains (socioeconomic hardship: aPOR 4.55, 95% CI 3.85–5.37; psychological burden: aOR 4.41, 95% CI 3.85–5.05; psychosocial burden: aOR 4.64, 95% CI 4.10–5.25) (all *p* < 0.001) (Table 2).

### Disability-type–specific associations with survivorship burden

In analyses restricted to mutually exclusive disability-type patterns **(**Table 3**)**, all disability types were independently associated with higher survivorship burden relative to survivors without disability. Compared with no disability, self-care disability only was associated with the highest odds of socioeconomic hardship (aPOR 2.63, 95% CI 2.12–3.27), followed by mobility disability only (aPOR 2.14, 95% CI 1.86–2.46) and cognitive disability only (aPOR 1.78, 95% CI 1.55–2.05).

**Table 3:** Adjusted associations between mutually exclusive disability-type patterns and multidimensional hardship domains

Panel A. Socioeconomic hardship (ordinal 0/1/2)				
Disability type	**Socioeconomic hardship** **aPOR (95% CI)**		**P-value**	
No disability	Ref		—	
Mobility only	2.14 (1.86–2.46)		< 0.001	
Cognitive only	1.78 (1.55–2.05)		< 0.001	
Self-care only	2.63 (2.12–3.27)		< 0.001	
Panel B. Psychological and psychosocial burden outcomes				
Disability type	**Psychological burden (frequent mental distress)** **aOR (95% CI)**	**Psychosocial burden (leisure-time physical inactivity)** **aOR (95% CI)**		**Global *p*-value**
No disability	Ref	Ref		—
Mobility only	1.66 (1.50–1.83)	2.98 (2.70–3.29)		< 0.001
Cognitive only	3.42 (3.02–3.87)	1.64 (1.45–1.85)		< 0.001
Self-care only	2.18 (1.85–2.57)	2.52 (2.13–2.97)		< 0.001

Abbreviations: *aOR*, adjusted odds ratio; *aPOR*, adjusted proportional odds ratio; *CI*, confidence interval; *BRFSS*, Behavioral Risk Factor Surveillance System

1. Exposure definition (mutually exclusive disability-type patterns): Disability-type categories were defined to be mutually exclusive based on three functional disability domains (mobility, cognitive, and self-care): no disability (reference), mobility only, cognitive only, and selfcare only

2. Outcome definitions:

• Socioeconomic hardship (ordinal 0/1/2): Sum of two indicators—(i) inability to work due to health and (ii) cost-related delay in medical care—categorized as 0, 1, or 2 hardships and modeled using ordinal logistic regression (aPOR)

• Psychological burden: Frequent mental distress, defined as ≥ 14 days of poor mental health in the past 30 days

• Psychosocial burden: Leisure-time physical inactivity, defined as no leisure-time exercise/physical activity

3. Modeling approach: Panel A reports aPORs from survey-weighted ordinal logistic regression models; Panel B reports aORs from survey-weighted logistic regression models. The reference group for all estimates is no disability

4. Covariate adjustment: All models were adjusted for all covariates listed in Table 1 , including age, sex, race/ethnicity, educational attainment, household income, marital status, residence (urban/rural), health insurance status, comorbidities (diabetes, heart disease, COPD, stroke, arthritis), primary care visit in the past year, treatment status, age at cancer diagnosis, state Medicaid expansion status, and cancer type

5. Global p-value: Global p-values were obtained from survey-adjusted Wald tests for the overall association between the categorical disability-type exposure and each outcome

6. Ordinal model assumption: The proportional odds assumption for ordinal logistic regression was assessed (and met)

Disability-specific patterns differed across psychological and psychosocial domains. Cognitive disability only demonstrated the strongest association with psychological burden (aOR 3.42, 95% CI 3.02–3.87), whereas mobility disability only was most strongly associated with psychosocial burden (aOR 2.98, 95% CI 2.70–3.29). Survivors with self-care disability only experienced elevated odds across both domains, including psychological burden (aOR 2.18, 95% CI 1.85–2.57) and psychosocial burden (aOR 2.52, 95% CI 2.13–2.97) (all *p* < 0.001) (Table 3).

### Medicaid expansion and disability-associated socioeconomic hardship

Among cancer survivors aged 18–64 years with household income < 25,000, socioeconomic hardship was consistently more prevalent among individuals with functional disability in both Medicaid expansion and non-expansion states (eTable 3). However, disability-associated socioeconomic hardship was attenuated in expansion states. For survivors with self-care disability, the hardship gap relative to survivors without disability was 18.7 percentage points in non-expansion states compared with 14.3 percentage points in expansion states, corresponding to a difference-in-differences estimate of − 4.4 percentage points (*p* = 0.03).

**Fig. 2:**
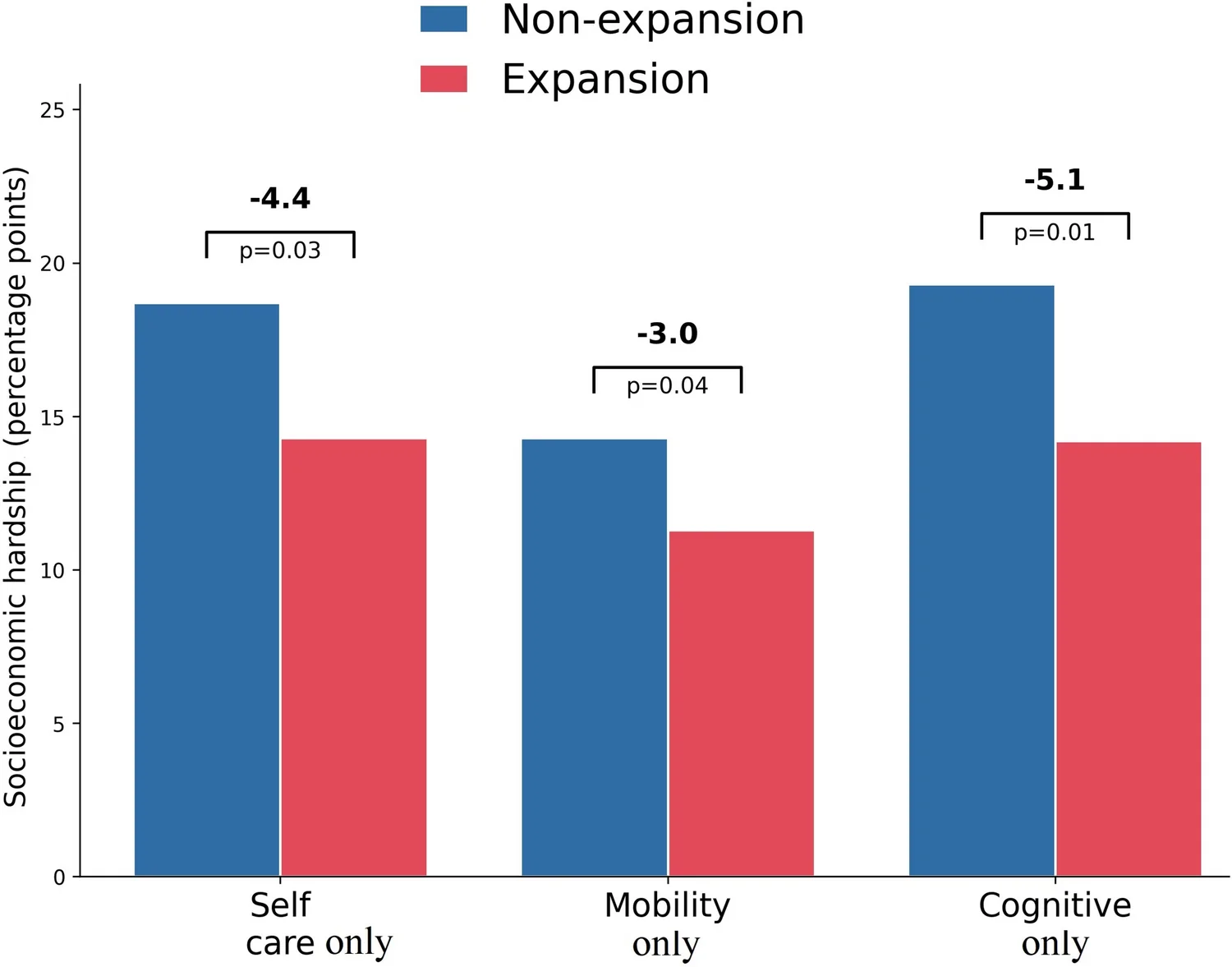
Socioeconomic hardship gaps by disability type and Medicaid expansion status. Bars depict the adjusted absolute differences (percentage points) in socioeconomic hardship between cancer survivors without and with disability within non–Medicaid expansion states (blue) and Medicaid expansion states (red), stratified by disability type (self-care, mobility, and cognitive). Brackets denote the difference-in-differences (DiD), calculated as the disability-associated hardship gap in expansion states minus the corresponding gap in non-expansion states, with DiD estimates shown above each pair of bars. *P*-values correspond to tests of the DiD estimates

Similar patterns were observed for mobility disability (14.3 vs 11.3 percentage points; difference-in-differences − 3.0, *p* = 0.04) and cognitive disability (19.3 vs 14.2 percentage points; difference-in-differences − 5.1, *p* = 0.01). These findings indicate that Medicaid expansion was associated with a modest but statistically significant reduction in disability-associated socioeconomic hardship among low-income cancer survivors (eTable 3) (Fig. 2).

### Sex differences in disability-related survivorship burden

Among female cancer survivors, any disability was associated with greater socioeconomic hardship severity (aPOR 2.62, 95% CI 2.30–2.98), higher odds of psychological burden, defined as frequent mental distress (aOR 2.71, 95% CI 2.43–3.02), and higher odds of psychosocial burden, defined as leisure-time physical inactivity (aOR 2.54, 95% CI 2.30–2.81) compared with women without disability (all *p* < 0.001). Among men, any functional disability was associated with higher odds of socioeconomic hardship, psychological burden, and psychosocial burden compared with no disability (aPOR 2.29; aORs 2.21 and 2.83, respectively; all *p* < 0.001).

Formal interaction testing demonstrated significant effect modification by sex. Disability-associated socioeconomic hardship (interaction aPOR 1.14, *p* = 0.002) and psychological burden (interaction aOR 1.23, *p* < 0.001) were more pronounced among women, whereas the association between disability and psychosocial burden was modestly weaker among women than men (interaction aOR 0.90, *p* = 0.01) (eTable 4).

## Discussion

As cancer survivorship continues to grow in the USA, attention has shifted towards quality of life among survivors, particularly in light of evidence that nearly two-thirds of US cancer survivors report difficulties with routine activities [3, 24]. Functional impairments in mobility, self-care, or cognition can substantially affect a survivor’s quality of life, by limiting daily functioning and contributing to various downstream hardships [25, 26]. The current study was important because it was the first nationally representative analysis to assess specific types of functional disability and their associations with survivorship hardship and burdens among US cancer survivors, using BRFSS data from 2017 to 2022. The data demonstrated that functional disabilities were differentially associated with distinct domains of survivorship burden, with mobility, cognitive and self-care limitations mapping onto psychosocial burdens, psychological burdens and socioeconomic hardships, respectively. Of note, sex-stratified analyses revealed that the magnitude and pattern of disability-associated burden differed between women and men, indicating important effect modification by sex. These findings highlight the multifaceted challenges faced by survivors living with disability and underscore the need to not only prolong cancer survival, but to actively support the well-being and daily functioning among individuals who survive.

Understanding how specific disability types shape survivorship burden is critical to guiding care and improving quality of life among cancer survivors. Of note, survivors with cognitive disability demonstrated the strongest association with psychological burden, as reflected by markedly higher rates of frequent mental distress, consistent with prior evidence that cancer-related cognitive impairment (“chemo brain”) exacerbates mood and anxiety disorders [27]. Clinically, these findings underscore the importance of routine cognitive and mental health screening, alongside interventions such as cognitive rehabilitation and counseling [28]. In contrast, survivors with mobility disability experienced disproportionate psychosocial and socioeconomic burden, including a higher likelihood of leisure time physical inactivity and inability to work, aligning with prior studies linking physical impairment to job loss, prolonged unemployment, and reduced activity [29]. Integrating physical therapy, tailored exercise programs, and vocational rehabilitation into survivorship care serves a dual goal of improving functional capacity and maintaining engagement in work and recreation [30]. Meanwhile, self-care disability was most strongly associated with socioeconomic hardship, including cost-related unmet medical needs and work limitations. Self-care impairment often reflects high dependency, with survivors requiring home health services, caregiving support, and assistive devices, thereby increasing out-of-pocket costs and financial strain [31]. Addressing these challenges requires coordinated care involving oncology, primary care, rehabilitation, social work, and home health services [32]. Overall, a “disability-informed” approach tailored to each cancer survivor’s functional status is critical towards advancing patient-centered care.

Understanding sex- and gender-related factors is essential to contextualize disability-associated disparities in survivorship burden, as these factors shape both the experience and consequences of functional limitations [33–35]. In the current study, disability was associated with higher socioeconomic hardship and psychological burden among both women and men; however, sex-stratified analyses revealed important differences in how disability translated into specific outcomes. Disability-related increases in socioeconomic hardship and psychological burden were more pronounced among female survivors, whereas disability was more strongly associated with psychosocial burden, defined as leisure-time physical inactivity, among male survivors. These patterns likely reflect the combined influence of biological and social mechanisms [36]. Prior survivorship research has noted that women report worse physical functioning and greater symptom burden than men, which may increase vulnerability to psychological distress and economic disruption when disability is present [33, 37]. Multidimensional financial toxicity frameworks further emphasize the reciprocal relationship between economic strain and mental health, supporting the interpretation that disability may be particularly destabilizing for women’s socioeconomic and psychological well-being [38, 39]. A growing proportion of cancer survivors experience financial toxicity [31]. In contrast, the stronger disability–inactivity association observed among men may reflect gendered norms of self-reliance and lower engagement with supportive services, creating pathways to behavioral disengagement as function declines [40, 41]. Underutilization of rehabilitation, delayed help-seeking, and limited uptake of structured exercise or supportive programs may allow functional impairments to translate more directly into inactivity, rather than being mitigated through early intervention [42, 43].

Beyond clinical care, these findings carry important policy implications. Among low-income, working-age cancer survivors, Medicaid expansion was associated with an attenuation of disability-associated socioeconomic hardship for survivors with self-care, mobility, and cognitive disabilities (eTable 3). Difference-in-differences analyses demonstrated smaller disability gaps in hardship in expansion versus non-expansion states, suggesting that insurance coverage can partially buffer disability-associated economic vulnerability. Nonetheless, substantial hardship persisted even in expansion states, underscoring that insurance coverage alone is insufficient to address the multidimensional needs of survivors living with functional disability [44, 45]. Many survivors require services—such as prolonged rehabilitation, durable medical equipment, mental health care, and home-based support—that remain variably covered or financially inaccessible despite insurance enrollment [46]. Notably, a recent national analysis reported that durable medical equipment (e.g., wheelchairs, canes, oxygen equipment) carried the highest out-of-pocket cost share among cancer survivors (39%), with utilization doubling between 1999 and 2018, reflecting persistent coverage limitations and administrative barriers that compound financial strain [47]. As such, routine functional assessments, analogous to distress screening, should be integrated into oncology and survivorship care to facilitate early identification of high-risk survivors and timely referral to supportive services [48]. Coverage policies should further ensure that multidisciplinary rehabilitation—including physical, occupational, cognitive, and psychosocial services—is treated as an essential benefit across Medicaid, Medicare, and private insurance plans [49]. Disability-inclusive survivorship models that embed rehabilitation specialists, social workers, and care coordinators within oncology teams, alongside survivorship plans addressing workplace and home accommodations, may help close persistent gaps [50]. Collectively, these findings support the need for coordinated insurance, clinical, and social policy approaches that move beyond coverage expansion alone to meaningfully reduce disability-related hardship among cancer survivors.

The findings of the current study should be interpreted in light of certain limitations. The use of survey-based data such as BRFSS limited the ability to incorporate detailed clinical information on cancer survivors, including cancer type, stage at diagnosis. In addition, BRFSS did not capture granular treatment timing or intensity (e.g., time since diagnosis, time since treatment completion, or specific treatment exposures), which limited the ability to distinguish individuals in active treatment from those further into survivorship and to assess whether specific therapies differentially shaped socioeconomic hardship, psychological burden, or psychosocial burden. This constraint was partially addressed by adjusting for self-reported treatment status (“treatment completed”) in all multivariable models; however, residual heterogeneity across survivorship phases may have persisted. Similarly, survey-based data may be susceptible to information bias compared with other methods of data collection. Nonetheless, the BRFSS dataset has been validated in multiple prior studies and has demonstrated reliability relative to other self-reported national health surveys [51]. Observational data are inherently prone to selection bias (i.e., the representativeness of the target population); however, this is generally mitigated in BRFSS through the use of raking methodology, which adjusts for sampling differences and enhances population-level generalizability [52]. Notwithstanding these limitations, the current study offers valuable insights into the relationship between functional disability and survivor hardship using a large, nationally representative dataset.

In conclusion, functional disability was a major driver of multidimensional survivorship burden among US cancer survivors with cognitive, self-care, and mobility limitations demonstrating distinct patterns of psychological, socioeconomic, and psychosocial hardship. Disability-related burdens differed meaningfully by sex, with women experiencing greater socioeconomic and psychological hardship and men exhibiting stronger associations with leisure time physical inactivity. Although Medicaid expansion modestly attenuated disability-associated socioeconomic hardship among low-income survivors, a substantial burden persisted. These findings underscore the need to embed routine functional screening, sex-sensitive survivorship care, and multidisciplinary rehabilitation into post-cancer care to preserve quality of life alongside survival.

## Supplementary Information

Below is the link to the electronic supplementary material.

ESM 1 (DOCX 31.5 KB)

## Data Availability

https://www.cdc.gov/brfss/annual_data/annual_2022.html

## References

[1] Cancer prevalence: how many people have cancer? https://www.cancer.org/cancer/survivorship/cancer-prevalence.html. Accessed June 13, 2025.

[2] Disparities in cancer survivorship | AACR. https://cancerprogressreport.aacr.org/disparities/cdpr24-contents/cdpr24-disparities-in-cancer-survivorship/. Accessed June 13, 2025.

[3] Study finds functional limitations increasing in survivors of cancer. https://www.hopkinsmedicine.org/news/newsroom/news-releases/2023/06/study-finds-functional-limitations-increasing-in-survivors-of-cancer. Accessed June 13, 2025.

[4] Cancer survivors have higher risk of functional disability- cancer therapy advisor. https://www.cancertherapyadvisor.com/news/cancer-survivors-functional-disability/. Accessed June 13, 2025.

[5] Schmidt JE, Beckjord E, Bovbjerg DH, et al. Prevalence of perceived cognitive dysfunction in survivors of a wide range of cancers: results from the 2010 LIVESTRONG survey. J Cancer Surviv. 2016;10(2):302–11. 10.1007/s11764-015-0476-5. PMC5772767 26238504

[6] Muhandiramge J, Orchard SG, Warner ET, van Londen GJ, Zalcberg JR. Functional decline in the cancer patient: a review. Cancers. 2022;14(6):1368. 10.3390/cancers14061368. PMC8946657 35326520

[7] Magasi S, Marshall HK, Winters C, Victorson D. Cancer survivors’ disability experiences and identities: a qualitative exploration to advance cancer equity. Int J Environ Res Public Health. 2022;19(5):3112. 10.3390/ijerph19053112. PMC8910238 35270802

[8] Tak HJ, Horner RD, Lee MS, Shih YCT. Impact of functional disability on health-care use and medical costs among cancer survivors. JNCI Cancer Spectr. 2023;7(5):pkad059. 10.1093/jncics/pkad059. PMC10505255 37584678

[9] CDC - BRFSS - BRFSS Statistical Briefs. https://www.cdc.gov/brfss/data_documentation/statistic_brief.htm. Published February 5, 2025. Accessed April 13, 2025.

[10] Behavioral Risk Factor Surveillance System. https://www.cdc.gov/brfss/index.html. Published June 4, 2025. Accessed June 15, 2025.

[11] Health-Related Quality of Life in Cancer... : Cancer. Ovid. 10.1002/cncr.23291

[12] Cao C, Yang L, Schmitz KH, Ligibel JA. Prevalence and cancer-specific patterns of functional disability among US cancer survivors, 2017–2022. J Clin Oncol. 2024;42(19):2257–70. 10.1200/JCO.23.02536. PMC11470834 38574313

[13] Communication with physicians about health care costs: survey of an insured population | The Permanente Journal. https://www.thepermanentejournal.org/doi/10.7812/TPP/16-070 . Accessed January 26, 2026. 10.7812/TPP/16-070 PMC5391781 28406786

[14] Yabroff KR, Zhao J, Zheng Z, Rai A, Han X. Medical financial hardship among cancer survivors in the United States: what do we know? What do we need to know? Cancer Epidemiol Biomarkers Prev. 2018;27(12):1389–97. 10.1158/1055-9965.EPI-18-0617. 30429132

[15] Blinder VS, Gany FM. Impact of cancer on employment. J Clin Oncol. 2020;38(4):302–9. 10.1200/JCO.19.01856. PMC6992498 31804857

[16] Tanner S, Engstrom T, Lee WR, et al. Mental health patient‐reported outcomes among adolescents and young adult cancer survivors: a systematic review. Cancer Med. 2023;12(17):18381–93. 10.1002/cam4.6444. PMC10524059 37596768

[17] Florin TA, Fryer GE, Miyoshi T, et al. Physical inactivity in adult survivors of childhood acute lymphoblastic leukemia: a report from the childhood cancer survivor study. Cancer Epidemiol Biomarkers Prev. 2007;16(7):1356–63. 10.1158/1055-9965.EPI-07-0048. 17627001

[18] Surveillance of demographic characteristics and health behaviors among adult cancer survivors — Behavioral Risk Factor Surveillance System, United States, 2009. https://www.cdc.gov/mmwr/preview/mmwrhtml/ss6101a1.htm. Accessed January 26, 2026. 22258477

[19] Associations of employment disruptions and financial hardship among individuals diagnosed with cancer in the US. | J Clin Oncol. https://ascopubs.org/doi/abs/10.1200/JCO.2023.41.16_suppl.12097 . Accessed January 26, 2026. 10.1158/2767-9764.CRC-23-0157 PMC10496757 37705562

[20] Material and psychological financial hardship related to employment disruption among female adolescent and young adult cancer survivors - Meernik - 2021 - Cancer - Wiley Online Library. https://acsjournals.onlinelibrary.wiley.com/doi/full/10.1002/cncr.33190 . Accessed January 26, 2026. 10.1002/cncr.33190 PMC7736150 33043464

[21] Ha DM, Prochazka AV, Bekelman DB, Stevens-Lapsley JE, Chan ED, Keith RL. Association of leisure-time physical activity with health-related quality of life among US lung cancer survivors. JNCI Cancer Spectr. 2021;5(1):pkaa118. 10.1093/jncics/pkaa118. PMC7853156 35075444

[22] Zhao G, Li C, Li J, Balluz LS. Physical activity, psychological distress, and receiving mental healthcare services among cancer survivors. J Cancer Surviv Res Pract. 2013;7(1):131–9. 10.1007/s11764-012-0254-6. PMC11250998 23184465

[23] Prevalence and cancer-specific patterns of functional disability among US cancer survivors, 2017–2022 | J Clin Oncol. https://ascopubs.org/doi/10.1200/JCO.23.02536 . Accessed June 13, 2025. 10.1200/JCO.23.02536 PMC11470834 38574313

[24] Cancer statistics, 2022.https://acsjournals.onlinelibrary.wiley.com/doi/epdf/10.3322/caac.21708?getft;integrator;mendeley;src;getftr;utm;source;mendeley Accessed June 13, 2025.

[25] Health care expenses and financial hardship among Medicare beneficiaries with functional disability | Health Policy | JAMA Network Open | JAMA Network. https://jamanetwork.com/journals/jamanetworkopen/fullarticle/2820090. Accessed June 13, 2025. 10.1001/jamanetworkopen.2024.17300 PMC11184460 38884997

[26] Fingerman KL, Ng YT, Huo M, Birditt KS, Charles ST, Zarit S. Functional limitations, social integration, and daily activities in late life. J Gerontol B Psychol Sci Soc Sci. 2021;76(10):1937–47. 10.1093/geronb/gbab014. PMC8598990 33460446

[27] Haywood D, Henry M, Dauer E, et al. Cancer-related cognitive impairment as a key contributor to psychopathology in cancer survivors: implications for prevention, treatment and supportive care. Support Care Cancer. 2024;32(7):480. 10.1007/s00520-024-08696-9. PMC11219369 38954104

[28] Duivon M, Lange M, Binarelli G, et al. Improve the management of cancer-related cognitive impairment in clinical settings: a European Delphi study. J Cancer Surviv. 2024;18(6):1974–97. 10.1007/s11764-023-01436-8. PMC11502546 37934312

[29] Cancer survivors and unemployment: a meta-analysis and meta-regression | Breast Cancer | JAMA | JAMA Network. https://jamanetwork.com/journals/jama/fullarticle/183387. Accessed June 13, 2025.

[30] Ma JK, Floegel TA, Li LC, et al. Tailored physical activity behavior change interventions: challenges and opportunities. Transl Behav Med. 2021;11(12):2174–81. 10.1093/tbm/ibab106. PMC8672936 34424344

[31] Medical financial hardship among cancer survivors in the United States - Zheng - 2019 - Cancer - Wiley Online Library. https://acsjournals.onlinelibrary.wiley.com/doi/10.1002/cncr.31913 . Accessed June 13, 2025. 10.1002/cncr.31913 30663039

[32] Leung DYP, Chan HYL, Chiu PKC, Lo RSK, Lee LLY. Source of social support and caregiving self-efficacy on caregiver burden and patient’s quality of life: a path analysis on patients with palliative care needs and their caregivers. Int J Environ Res Public Health. 2020;17(15):5457. 10.3390/ijerph17155457. PMC7432213 32751147

[33] Tinsley-Vance SM, Mertilus DSD, Nodzon L, Lengacher CA. An integrative review of sex differences in quality of life and symptoms among survivors of hematologic malignancies. Oncol Nurs Forum. 2023;50(3):299–312. 10.1188/23.ONF.299-312. PMC11401479 37155973

[34] Praça MSL, de Sousa FTR, Cândido EB, Lamaita RM, Wender MCO, Silva AL. Beyond the diagnosis: gender disparities in the social and emotional impact of cancer. Rev Assoc Médica Bras. 70(Suppl 1):e2024S115. 10.1590/1806-9282.2024S115 PMC11164259 38865535

[35] Disparities in Cancer Survivorship | AACR. https://cancerprogressreport.aacr.org/disparities/cdpr24-contents/cdpr24-disparities-in-cancer-survivorship/. Accessed January 26, 2026.

[36] Göttgens I, Oertelt-Prigione S. Gender as a contextual factor in quality of life of cancer survivors: a literature review. Oncol Res Treat. 2025;48(1–2):48–56. 10.1159/000543067. 39675350

[37] Oertelt-Prigione S, de Rooij BH, Mols F, et al. Sex-differences in symptoms and functioning in >5000 cancer survivors: results from the PROFILES registry. Eur J Cancer. 2021;156:24–34. 10.1016/j.ejca.2021.07.019. 34411849

[38] Dee EC, Nipp RD, Muralidhar V, et al. Financial worry and psychological distress among cancer survivors in the United States, 2013-2018. Support Care Cancer. 2021;29(9):5523–35. 10.1007/s00520-021-06084-1. 33725174

[39] Benedict C, Fisher S, Schapira L, et al. Greater financial toxicity relates to greater distress and worse quality of life among breast and gynecologic cancer survivors. Psychooncology. 2022;31(1):9–20. 10.1002/pon.5763. PMC9809212 34224603

[40] DeGuzman PB, Vogel DL, Bernacchi V, Scudder MA, Jameson MJ. Self-reliance, social norms, and self-stigma as barriers to psychosocial help-seeking among rural cancer survivors with cancer-related distress: qualitative interview study. JMIR Form Res. 2022;6(5):e33262. 10.2196/33262. PMC9164097 35588367

[41] The role of need for control and self‐reliance in gender and age differences in bowel cancer screening participation - Goodwin - 2022 - Psycho-Oncology - Wiley Online Library. https://onlinelibrary.wiley.com/doi/10.1002/pon.5979 . Accessed January 26, 2026. 10.1002/pon.5979 35726402

[42] Galvão DA, Chambers SK. Exercise medicine in men with prostate cancer: breaking barriers to increase participation. Prostate Cancer Prostatic Dis. 2021;24(4):942–3. 10.1038/s41391-021-00406-4. 34145428

[43] Program Improves Physical Activity in Cancer Survivors - NCI. https://www.cancer.gov/news-events/cancer-currents-blog/2024/cancer-survivors-minority-underserved-improving-activity. Published July 3, 2024. Accessed January 26, 2026.

[44] Williams CP, Rocque GB, Caston NE, et al. Health insurance and financial hardship in cancer survivors during the COVID-19 pandemic. PLoS One. 2022;17(8):e0272740. 10.1371/journal.pone.0272740. PMC9355233 35930603

[45] Tarazi WW, Bradley CJ, Harless DW, Bear HD, Sabik LM. Medicaid expansion and access to care among cancer survivors: a baseline overview. J Cancer Surviv. 2016;10(3):583–92. 10.1007/s11764-015-0504-5. PMC4864046 26662864

[46] Smith GL, Mendoza TR, Lowenstein LM, Shih YCT. Financial hardship in survivorship care delivery. J Natl Cancer Inst Monogr. 2021;2021(57):10–4. 10.1093/jncimonographs/lgaa012. PMC8415532 34478512

[47] Cancer survivors’ use of medical equipment increasing despite high OOP costs - Cancer Therapy Advisor. https://www.cancertherapyadvisor.com/news/cancer-survivors-use-of-medical-equipment-increasing-despite-high-oop-costs/. Accessed January 26, 2026.

[48] Survivorship care for people affected by advanced or metastatic cancer: MASCC-ASCO standards and practice recommendations | JCO Oncol Pract. https://ascopubs.org/doi/10.1200/OP.23.00716 . Accessed January 26, 2026. 10.1200/OP.23.00716 38684036

[49] Mollica MA, McWhirter G, Tonorezos E, et al. Developing national cancer survivorship standards to inform quality of care in the United States using a consensus approach. J Cancer Surviv. 2024;18(4):1190–9. 10.1007/s11764-024-01602-6. PMC11324674 38739299

[50] Jefford M, Howell D, Li Q, et al. Improved models of care for cancer survivors. Lancet. 2022;399(10334):1551–60. 10.1016/S0140-6736(22)00306-3. PMC9009839 35430022

[51] Pierannunzi C, Hu SS, Balluz L. A systematic review of publications assessing reliability and validity of the Behavioral Risk Factor Surveillance System (BRFSS), 2004-2011. BMC Med Res Methodol. 2013;13:49. 10.1186/1471-2288-13-49. PMC3622569 23522349

[52] Sedani AE, Frank-Pearce SG, Beebe LA, et al. Cannabis use and subsequent cigarette discontinuation among U.S. adults in the Population Assessment of Tobacco and Health Study, waves 1–5. Nicotine Tob Res. 2024;27(2):208–16. 10.1093/ntr/ntae202. PMC11879569 39178320

