# Supplementary material for: Functional disability and multidimensional hardship among cancer survivors in the USA: a nationally representative study
Source: J Cancer Surviv. 2026 Feb 12;20(Suppl 2):1525–35. doi: 10.1007/s11764-026-01981-y (PMC13744518; doi:10.1007/s11764-026-01981-y)
Supplement: Supplementary file 1 [file 11764_2026_1981_MOESM1_ESM.docx]

**Supplemental Online Content**

**eTable 1.** Baseline sociodemographic, socioeconomic, insurance, and disability characteristics of cancer survivors compared with the general U.S. adult population in the unweighted BRFSS sample.

**eTable 2.** Distribution of mutually exclusive functional disability-type patterns among cancer survivors in the weighted analytic cohort.

**eTable 3**. Socioeconomic hardship by disability type and Medicaid expansion status among low-income, working-age cancer survivors (18–64 years), including difference-in-differences estimates.

**eTable 4.** Sex-stratified adjusted associations between disability burden and multidimensional survivorship outcomes, including socioeconomic hardship and psychological and psychosocial burdens.

**eTable 1.** Baseline sociodemographic, socioeconomic, insurance, and disability characteristics of cancer survivors compared with the general U.S. adult population in the unweighted BRFSS sample.

| **Characteristics** | **Cancer survivors**  **(n= 26 864, 1.0%)** | **General population**  **(n = 2 564 639, 99.0%)** | **p-value^1^** |
| --- | --- | --- | --- |
| **Age (y) Median [IQR]** | 62 [53–73] | 58 [41-69] | <0.001 |
| **Age, n (%)** |  |  | <0.001 |
| < 65 years | 8,704 (32.4) | 1 618 970 (63.0) |  |
| ≥ 65 years | 18,160 (66.4) | 897 465 (35.1) |  |
| **Sex, n (%)** |  |  | <0.001 |
| Male | 9,241 (34.4) | 1,184,863 (46.2) |  |
| Female | 17,622 (65.6) | 1,379,775 (53.8) |  |
| **Ethnicity, n (%)** |  |  | <0.001 |
| White | 21,840 (81.3) | 1,940,948 (75.7) |  |
| Black | 1,746 (6.5) | 200,041 (7.8) |  |
| Asian | 483 (1.8) | 64,115 (2.5) |  |
| Native American | 537 (2.0) | 41,034 (1.6) |  |
| Hispanic | 1424 (5.3) | 248,769 (9.7) |  |
| Other | 806 (3.0) | 69,245 (2.7) |  |
| **Area, n (%)** |  |  | <0.001 |
| Urban | 15,930 (59.3) | 1,872,186 (73.0) |  |
| Rural | 7,092 (26.4) | 692,452 (23.0) |  |
| **Education, n (%)** |  |  | <0.001 |
| < High school | 1, 558(5.8) | 182,089 (7.1) |  |
| High school/some college | 14,640 (54.5) | 1,379,775 (53.8) |  |
| ≥ College degree | 10,638 (39.6) | 1,002,773 (39.1) |  |
| **Annual household income ($), n (%)** |  |  | <0.001 |
| < 25 000 | 3,331 (12.4) | 348,790 (13.6) |  |
| 25 000–74 999 | 5,399 (20.1) | 556,526 (21.7) |  |
| > 75 000 | 3,456 (12.9) | 477,022 (18.6) |  |
| **Marital status, n (%)** |  |  | <0.001 |
| Married | 13, 728 (51.1) | 1,246,414 (48.6) |  |
| Not married | 13,136 (49.9) | 1,318,224 (51.3) |  |
| **Disability, n (%)** |  |  | <0.001 |
| Self-care disability | 1,719 (6.4) | 102,585 (4.0) |  |
| Mobility disability | 7,522 (28.0) | 407,777 (15.9) |  |
| Cognitive disability | 3,465 (12.9) | 269,287 (10.5) |  |

**^Abbreviations:^** ^BRFSS, Behavioral Risk Factor Surveillance System; IQR, interquartile range.^

1. ^Cancer survivors were identified by self-reported history of any cancer diagnosis excluding non-melanoma skin cancer.^
2. ^Estimates are based on the unweighted BRFSS sample and are presented as counts (percentages) unless otherwise specified.^
3. ^Disability domains were defined using standard BRFSS items:^
   - *^Mobility disability:^* ^serious difficulty walking or climbing stairs^
   - *^Cognitive disability:^* ^serious difficulty concentrating, remembering, or making decisions^
   - *^Self-care disability:^* ^difficulty dressing or bathing^
4. ^P-values were derived using chi-square tests for categorical variables and the Kruskal–Wallis test for continuous variables.^
5. ^All statistical tests were two-sided, with significance set at p < 0.05.^

**eTable 2.** Distribution of mutually exclusive functional disability-type patterns among cancer survivors

| **Disability-type pattern** | **Weighted N** | **Weighted %** |
| --- | --- | --- |
| **No disability** | 2,012,487 | 56.4% |
| **Mobility only** | 445,912 | 12.5% |
| **Cognitive only** | 300,418 | 8.4% |
| **Self-care only** | 171,034 | 4.8% |
| **≥2 disability domains** | 640,667 | 17.9% |

**Abbreviations**: BRFSS, Behavioral Risk Factor Surveillance System.

1. Disability-type patterns were defined using three functional disability domains (mobility, cognitive, and self-care) and categorized as mutually exclusive.
2. “Mobility only,” “cognitive only,” and “self-care only” indicate reporting exactly one disability domain.
3. “≥2 disability domains” includes respondents reporting two or more disability domains.
4. Estimates are weighted to represent the U.S. population of cancer survivors.

**eTable 3.** Socioeconomic hardship by disability type and Medicaid expansion status among low-income, working-age cancer survivors (18–64 years), including difference-in-differences estimates.

| **Disability type** | **Hardship risk**  **Non expansion (%) (No → Yes)** | **Hardship risk**  **Expansion (%)**  **(No → Yes)** | **Disability Gap Non-expansion (%)** | **Disability Gap Expansion (%)** | **Difference-in-Differences**  **(p-value)*** |
| --- | --- | --- | --- | --- | --- |
| **Self-care** | 21.4 → 40.1 | 18.9 → 33.2 | +18.7 | +14.3 | −4.4 (p = 0.03) |
| **Mobility** | 17.6 → 31.9 | 15.4 → 26.7 | +14.3 | +11.3 | −3.0 (p = 0.04) |
| **Cognitive** | 19.1 → 38.4 | 16.8 → 31.0 | +19.3 | +14.2 | −5.1 (p = 0.01) |

**Abbreviations:** DiD, difference-in-differences.

1. Analyses were restricted to low-income cancer survivors aged 18–64 years.
2. Socioeconomic hardship was defined as reporting ≥1 of the following indicators:
   (i) inability to work due to health, or
   (ii) cost-related delay in needed medical care.
3. Medicaid expansion status was classified according to state-level implementation under the Affordable Care Act during the study period.
4. Disability gap represents the difference in hardship prevalence between survivors with the specified disability type and those without disability.
5. Difference-in-differences estimates reflect the net change in disability-associated hardship attributable to Medicaid expansion.
6. Statistical significance was assessed using two-sided tests with p < 0.05.

**eTable 4.** Sex-stratified adjusted associations between disability burden and multidimensional survivorship outcomes, including socioeconomic hardship and psychological and psychosocial burdens.

| **Panel A. Female cancer survivors** | | |
| --- | --- | --- |
| **Outcome** | **Model** | **Any disability vs none** |
| **Socioeconomic hardship (0/1/2)** | Ordinal logit (aPOR) | 2.62 (2.30–2.98) |
| **Psychological burden (FMD)** | Logit (aOR) | 2.71 (2.43–3.02) |
| **Psychosocial burden (inactivity)** | Logit (aOR) | 2.54 (2.30–2.81) |
| **Panel B. Male cancer survivors** | | |
| **Outcome** | **Model** | **Any disability vs none** |
| **Socioeconomic hardship (0/1/2)** | Ordinal logit (aPOR) | 2.29 (2.01–2.60) |
| **Psychological burden (FMD)** | Logit (aOR) | 2.21 (1.97–2.49) |
| **Psychosocial burden (inactivity)** | Logit (aOR) | 2.83 (2.55–3.13) |
| **Panel C. Sex interaction (effect modification)** | | |
| **Outcome** | **Female × disability interaction** | **P-value** |
| **Socioeconomic hardship (0/1/2)** | 1.14 | 0.002 |
| **Psychological burden (FMD)** | 1.23 | <0.001 |
| **Psychosocial burden (inactivity)** | 0.90 | 0.01 |

**Abbreviations:** aOR, adjusted odds ratio; aPOR, adjusted proportional odds ratio; CI, confidence interval; FMD, frequent mental distress; BRFSS, Behavioral Risk Factor Surveillance System.

1. “Any disability” indicates reporting ≥1 functional disability domain (mobility, cognitive, or self-care), with no disability as the reference group.
2. **Outcome definitions:**
   - *Socioeconomic hardship* was defined as the sum of two indicators—(i) inability to work due to health and (ii) cost-related delay in medical care—categorized as 0, 1, or 2 hardships and modeled using ordinal logistic regression (aPOR).
   - *Psychological burden* was defined as frequent mental distress (≥14 days of poor mental health in the past 30 days).
   - *Psychosocial burden* was defined as leisure-time physical inactivity (no leisure-time physical activity).
3. Panels A and B present sex-stratified, survey-weighted regression models. Panel C reports multiplicative interaction terms between sex and disability status.
4. All models were adjusted for covariates listed in Table 1, including age, race/ethnicity, educational attainment, household income, marital status, urban/rural residence, health insurance status, comorbidities (diabetes, heart disease, COPD, stroke, arthritis), primary care visit in the past year, treatment status, age at cancer diagnosis, state Medicaid expansion status, and cancer type.
5. All analyses incorporated BRFSS survey weights and design variables to generate nationally representative estimates.
6. All p-values are two-sided
